# CAN PERSONS WITH DEMENTIA MEANINGFULLY PARTICIPATE IN ADVANCE CARE PLANNING CONVERSATIONS? A MIXED-METHODS STUDY

**DOI:** 10.1093/geroni/igz038.209

**Published:** 2019-11-08

**Authors:** Mi-Kyung Song, Sandra Ward, Ken Hepburn, Sudeshna Paul, Hyejin Kim, Darby Morhardt, Raj C Shah

**Affiliations:** 1 Nell Hodgson Woodruff School of Nursing, Emory University, Atlanta, Georgia, United States; 2 University of Wisconsin-Madison, Madison, Wisconsin, United States; 3 Emory University, Atlanta, Georgia, United States; 4 Emory School of Nursing, Atlanta, Georgia, United States; 5 Northwestern University, Chicago, Illinois, United States; 6 Rush University, Chicago, Illinois, United States

## Abstract

Studies of advance care planning (ACP) in persons living with dementia (PLWDs) are rare. We conducted an intervention development study to adapt an efficacious ACP intervention, SPIRIT (Sharing Patient’s Illness Representations to Increase Trust), for PLWDs in early stages and their surrogates and assessed the feasibility/acceptability of the adapted SPIRIT. SPIRIT was adapted by the investigators and underwent expert panel review. The refined SPIRIT was then evaluated in a randomized trial with 23 dyads of PLWDs and their surrogates. Dyads were randomized to SPIRIT in-person (in a private room in a memory clinic) or SPIRIT remote (via videoconferencing from home). Participants completed preparedness outcome measures (dyad congruence on goals of care, patient decisional conflict, surrogate decision-making confidence) 2-3 days postintervention along with a semi-structured interview. PLWDs’ levels of articulation of end-of-life wishes during SPIRIT sessions were rated (3 = expressed wishes very coherently, 2 = somewhat coherently, 1 = unable to express wishes coherently). Fourteen PLWDs had moderate dementia, but all 23 were able to articulate their end-of-life wishes very or somewhat coherently during the SPIRIT session. While decision-making capacity was higher in PLWDs who articulated their wishes very coherently, global cognitive function did not differ by articulation levels. PLWDs and surrogates perceived SPIRIT as beneficial, but the preparedness outcomes did not change from baseline to postintervention in either group. SPIRIT for PLWDs and surrogates engaged them in meaningful ACP discussions. Further research is warranted to test its efficacy and long-term outcomes with a larger and diverse sample.

